# Enhanced inhibitory synaptic transmission in the spinal dorsal horn mediates antinociceptive effects of TC-2559

**DOI:** 10.1186/1744-8069-7-56

**Published:** 2011-08-04

**Authors:** Long-Zhen Cheng, Lei Han, Jing Fan, Lan-Ting Huang, Li-Chao Peng, Yun Wang

**Affiliations:** 1 Institutes of Brain Science and State Key Laboratory of Medical Neurobiology, Fudan University, Shanghai 200032, China; 2 The Department of Anaesthesia, The Fifth People's Hospital of Shanghai, Fudan University, Shanghai 200240, China

**Keywords:** TC-2559, α4β2 nAChRs, Formalin test, CCI, sIPSCs, Pain, Spinal cord slice

## Abstract

**Background:**

TC-2559 is a selective α4β2 subtype of nicotinic acetylcholine receptor (nAChR) partial agonist and α4β2 nAChR activation has been related to antinociception. The aim of this study is to investigate the analgesic effect of TC-2559 and its underlying spinal mechanisms.

**Results:**

1) *In vivo *bioavailability study: TC-2559 (3 mg/kg) had high absorption rate in rats with maximal total brain concentration reached over 4.6 μM within first 15 min after administration and eliminated rapidly with brain half life of about 20 min after injection. 2) *In vivo *behavioral experiments: TC-2559 exerts dose dependent antinociceptive effects in both formalin test in mice and chronic constriction injury (CCI) model in rats by activation of α4β2 nAChRs; 3) Whole-cell patch-clamp studies in the superficial dorsal horn neurons of the spinal cord slices: perfusion of TC-2559 (2 μM) significantly increased the frequency, but not amplitude of spontaneous inhibitory postsynaptic currents (sIPSCs). The enhancement of sIPSCs was blocked by pre-application of DHβE (2 μM), a selective α4β2 nicotinic receptor antagonist. Neither the frequency nor the amplitude of spontaneous excitatory postsynaptic currents (sEPSCs) of spinal dorsal horn neurons were affected by TC-2559.

**Conclusions:**

Enhancement of inhibitory synaptic transmission in the spinal dorsal horn via activation of α4β2 nAChRs may be one of the mechanisms of the antinociceptive effects of TC-2559 on pathological pain models. It provides further evidence to support the notion that selective α4β2 subtype nAChR agonist may be developed as new analgesic drug for the treatment of neuropathic pain.

## Background

Opioids and the nonsteroidal anti-inflammatory drugs (NSAIDs) are the two classes of most common used analgesic drugs. Due to the deficiencies associated with these two classes of compounds, exploration of novel targeted drugs for pain relief has been the interests to many researchers [1-3].

Nicotine, agonist of nicotinic acetylcholine receptors (nAChRs), has long been known to have antinociceptive effects, but failed to be developed as an analgesic agent because of its relatively modest antinociceptive effects and toxicities associated with nicotine [4-7]. In addition, epibatidine, a non-selective nAChR agonist, is the most potent pain relief ligand known so far, but with server nicotinic type side effects [8]. Thus it will be necessary to develop a subtype selective ligand to get an acceptable therapeutic index. There are at least 12 different subunits of neuronal nAChRs, including α2 ~ α10 and β2 ~ β4 have been identified so far and these subunits form many different subtypes of nAChRs with pentameric structures consisting of homomers or heteromers [9]. Homomeric nAChRs are assembles with α subunits, while heteromeric nAChRs comprise various combinations of α and β subunits [10,11]. However, the analgesic effects of nAChRs related to α4β2 subtype has only been studied with the aid of the selective antagonists [11,12], but the direct analgesic effect of the selective agonist of the α4β2 subtype of nAChR has not been reported so far, due to lack of selective agonist commercially available. Recently, TC-2559 has been developed as a partial neuronal nicotinic receptor agonist [13] and later been further profiled and characterized as a selective agonist at α4β2 subtype of nAChRs both *in vitro *[14] and *in vivo *[14-16]. TC-2559 has an *in vitro *EC_50 _of 100 nM on α4β2-transfected HEK cells, ineffective on α7 and α3β2 receptors up to 10 μM, and weakly effective on α2β4, α4β4 and α3β4 receptors, with EC_50_'s in the range of 10-30 μM [14]. Our previous studies also demonstrated *in vivo *that TC-2559, by selectively activation of α4β2 subtype of nAChRs, enhanced dopaminergic neuronal firing and suppressed of long term potentiation of hippocampal dentate gyrus neurons in anaesthetized rats [14-16].

In current study, a combination of *in vivo *animal pathological pain models and *in vitro *whole-cell patch-clamp recordings in the spinal cord slices were used to examine the antinociceptive efficiency of TC-2559 and its underling spinal mechanism. Our results demonstrated that TC-2559 effectively inhibited pathological pain in animal neuropathic pain models and enhanced the inhibitory neurotransmission, but not excitatory transmission, in the spinal dorsal horn via activation of α4β2 subtype of nicotinic receptors.

## Results

### Brain exposure results of TC2559

**Figure 1:**
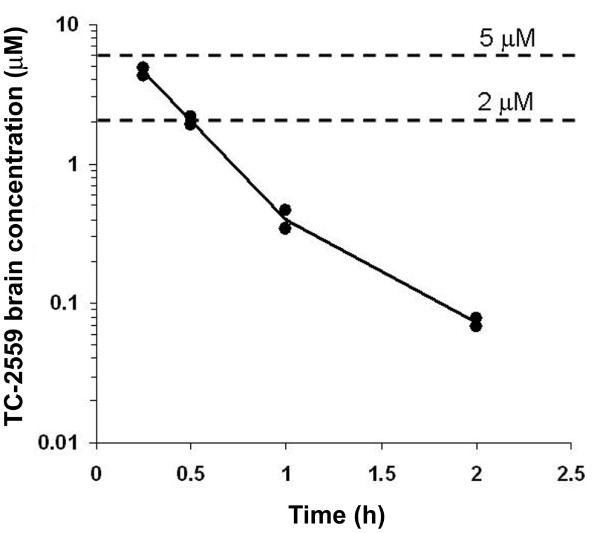
**The brain bioavailability of TC-2559 after systemic administration in rat**. Trace showing total brain concentration of TC-2559 (3 mg/kg, i.p.) at the time point from 0.25 to 2 h after i.p. injection (The line represented the average volumes from two animals).

In order to identify the appropriate systemic dose for the *in vivo *behavioral pain model studies, TC-2559 used in the present study was initially investigated for brain bioavailability. Bolus dose of TC-2559 (3 mg/kg) was injected i.p. and then the total brain concentrations were measured at 0.25, 0.5, 1 and 2 hours after administration. TC-2559 was rapidly absorbed following i.p. administration, with maximum brain concentrations of about 4.6 μM achieved 15 minutes after dosing. The clearance of the compound from brain was very rapid, with a half life of about 20 minutes and almost totally eliminated about 2 h after injection (Figure 1). This result is very similar to that we previously reported by intravenous TC-2559 administration [16]. Thus, the brain (as well as spinal cord) concentration of TC-2559 achieved by i.p. administration of 3 mg/kg dose is sufficient for activation of α4β2 subtype of nicotinic receptors.

### TC-2559 dose dependently reduced acute formalin-induced biphasic nociceptive responses in mice

The formalin test for nociception, which is widely used and easier to perform, involves moderate, continuous pain generated by injured tissue. It is a useful model, particularly for the screening of novel compounds, since it encompasses inflammatory, neurogenic, and central mechanisms of nociception [17,18]. Thus the effect of TC-2559 on formalin test was first examined.

**Figure 2:**
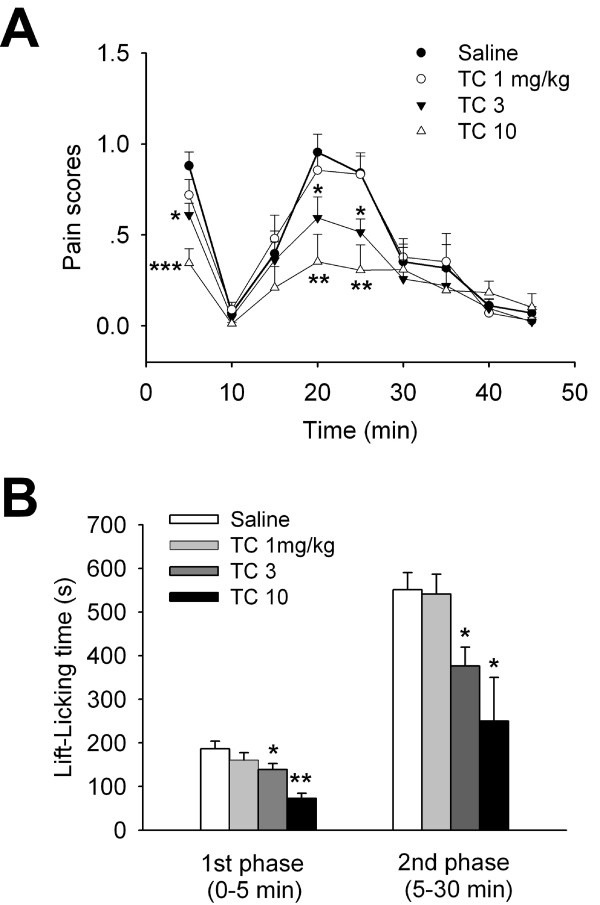
**Effect of TC-2559 on formalin-induced biphasic nociceptive responses in mice**. ***A***, Traces showing intraperitoneal injection of TC-2559 (1, 3, 10 mg/kg) dose dependently suppressed formalin induced progressive biphasic pain behavior score; ***B***, Bar histogram showing the group data of antinociceptive effect of TC-2559 on formalin induced paw lift-licking time in both early 1st phase (0-5 min) and late 2nd phase (5-30 min) of mice formalin model. *P < 0.05, **P < 0.01 compared with vehicle saline control (two-way ANOVA followed by the Holm-Sidak test).

Unilateral intraplantar injection of diluted formalin (5%) induced characteristic biphasic nociceptive behaviour responses: the first phase lasted for ~5 min and the second phase was between 10 to 40 min (Figure 2A), similar as we previously reported [19]. TC-2559 (1, 3, 10 mg/kg, i.p.) dose dependently reduced both early and late phases of formalin induced nociceptive behavioral responses (Figure 2A). In compared to the saline control group (n = 6), TC-2559 had no significant antinociceptive effect at low dose (1 mg/kg, n = 5), but significantly inhibited formalin induced pain behavior at higher doses tested (3 and 10 mg/kg, n = 5 for both groups) (*P *< 0.001, two-way ANOVA) (Figure 2B). These results demonstrated that TC-2559 has dose dependent analgesic property in mouse formalin pain models.

### TC-2559 dose dependently inhibited CCI-induced neuropathic pain in rats

**Figure 3:**
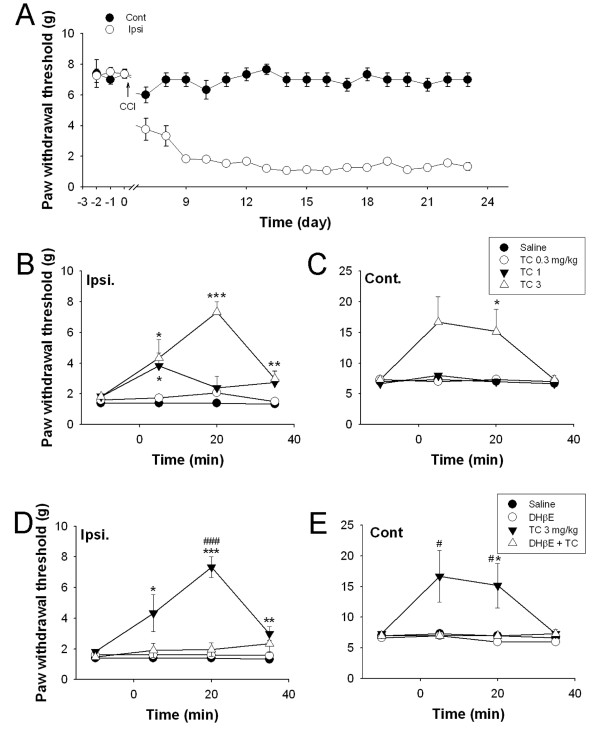
**TC-2559 dose dependently reduced CCI-induced neuropathic pain in rats**. ***A***, chart showing the progressive change of the paw withdraw threshold in both ipsilateral and contralateral hind paw before and after CCI injury. Note the enhanced mechanical allodynia in ipsilateral hind paw but not the contralateral uninjured hind paw. ***B-C***, Traces showing TC-2559 (i.p.) dose dependently reversed CCI-induced mechanical allodynia in both ipsilateral (B) and contralateral (C) hind paws of CCI rats (n = 6 for each group). Note: at 1 mg/kg dose, TC-2559 only inhibited the mechanical allodynia in CCI hind paw (ipsilateral). ***D-E***, Traces showing DHβE (2 mg/kg, i.p.) reversed TC-2559 (3 mg/kg, i.p.) induced mechanical allodynia inhibition in both ipsilateral and contralateral hind paws of the CCI rats (n = 6 for each group). *P < 0.05, **P < 0.01 and ***P < 0.001 compared with saline. ^#^*P *< 0.05 and ^###^*P *< 0.001 compared with 3 mg/kg TC-2559. (two-way ANOVA followed by the Holm-Sidak test).

The chronic constriction injury (CCI) model is one of the best-characterized behavioral models for neuropathic pain in rats [20]. To further examine whether TC-2559 contributes to the neuropathic pain, TC-2559 was tested on rat CCI models. Consistent with a previous study [19], unilateral CCI operation induced robust mechanical allodynia, reached mechanical sensitivity of lower than 2 g, between 7 to 9 days on the ipsilateral hind paw, but without effect on the contralateral hindpaw in current study (Figure 3A). The rats with no allodynia (n = 38) on ipsilatral hindpaw were discarded from the further pharmacological study. The allodynia induced by CCI were persisted for weeks in the ipsilateral hindpaw, as shown in Figure 3A (*P *< 0.001, n = 6).

Drugs were tested on rats with stable allodynia (mechanical sensitivity lower than 2 g) after CCI. Baseline mechanical sensitivity was measured 10 min before drug injection and saline was served as vehicle control. On ipsilateral hind paw, TC-2559 (i.p.) dose dependently reversed CCI induced allodynia in comparison with saline control. TC-2559 at doses of 1 mg/kg (n = 6) and 3 mg/kg (n = 6) both significantly (*P *< 0.001 and *P *< 0.001, respectively) reversed CCI induced the paw withdrawal threshold decreases (Figure 3B), while TC-2559 at 0.3 mg/kg had no significant effect (n = 6, *P *= 0.051). The analgesic effect of TC-2559 was rapid onset with the maximal response proximally at 5 and 20 min after administration for 1 mg/kg and 3 mg/kg doses, respectively, and the effect was also short lasting, lasted for less than 40 min after injection (Figure 3B), which was consistent with our bioavailability study.

In contrast, in the contralateral hind paw, TC-2559 had no effect on the sensory response threshold at the mid (1 mg/kg, i.p.; *P *= 0.772, n = 6) and lower (0.3 mg/kg, i.p.; *P *= 0.591, n = 6) doses tested, but significantly enhanced the paw withdrawal threshold to above the control level while given (i.p.) with higher dose at 3 mg/kg (*P *= 0.002, n = 6) (Figure 3C).

In order to confirm that the analgesic effect of TC-2559 was due to activation of α4β2 nAChR subtype, the selective α4β2 subtype antagonist DHβE was employed. Pretreatment with DHβE (2 mg/kg, i.p.) fully inhibited the analgesic effect induced by 3 mg/kg TC-2559 on both inpsi- and contra-lateral hind paw of CCI rats (*P *< 0.001 and *P *= 0.003, respectively; Two-way ANOVA; n = 6 in either groups) (Figure 3D, E). DHβE alone had no effect on the allodynia induced by CCI (*P *= 0.113, n = 6) (Figure 3E). These results indicate that TC-2559, by activation of α4β2 subtype of nAChRs, has strong antinociceptive properties on CCI-induced mechanical allodynia.

### TC-2559 enhanced inhibitory synaptic transmission by activation of α4β2 subtype of nAChRs in superficial dorsal horn neurons

To examine the mechanism underlies the antinociceptive effects of TC-2559, whole-cell patch-clamp recordings of superficial dorsal horn neurons in the spinal cord slices were performed to investigate whether activation of α4β2 nAChR would cause enhanced general inhibition and/or reduced excitatory pain signal transmission in dorsal horn neurons, in which nociceptive information is modulated and conveyed to projection neurons.

We first examined the effects of TC-2559 on inhibitory synaptic transmission of spontaneous inhibitory postsynaptic currents (sIPSCs). DNQX (15 μM) was continueously perfused to block non-NMDA receptor glutamatergic currents. Membrane potential was held at -70 mV and the IPSCs were observed as inward currents due to the chloride-based intra-pipette solution. sIPSCs were observed in approximately 88% (n = 101/115) neurons tested.

**Figure 4:**
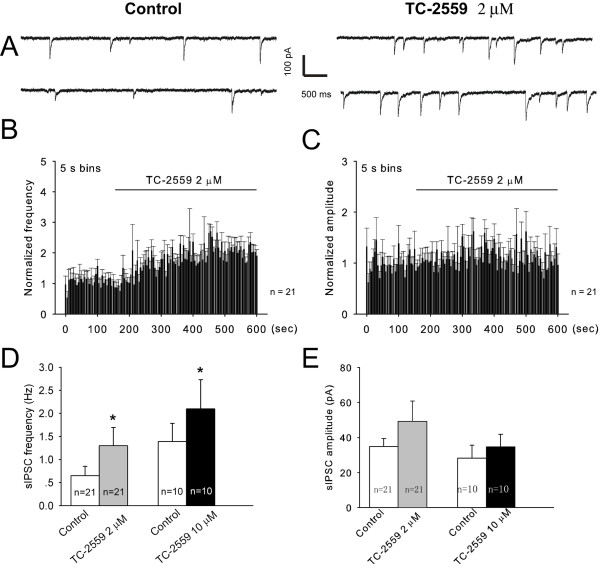
**TC-2559 enhanced the frequency of sIPSCs in the superficial dorsal horn of the spinal cord slices**. ***A***, Whole-cell Patch-clamp recording of sIPSCs in a substantia gelatinosa (SG) neuron in the presence of 15 μM DNQX before and during bath application of 2 μM TC-2559. The holding voltage is -70 mV. ***B-C***, Normalized rate histogram showing the sIPSC frequency (B) and amplitude (C) before and during bath application of 2 μM TC-2559. ***D-E***, Application of TC-2559 (2, 10 μM) significantly enhanced the frequency of sIPSCs, but not the amplitude. *P < 0.05 (paired *t *test).

As shown in Figure 4, perfusion of TC-2559 (2 μM) resulted in a rapid and significant increases in sIPSC frequency in majority of the neurons (18 out of 21, characterized as at least 20% increase of the sIPSC frequency to above the baseline activities, see method section) tested. As a whole group, TC-2559 increased the sIPSC frequency from 0.65 ± 0.20 Hz of the baseline control to 1.30 ± 0.39 Hz during TC-2559 perfusion (*P *= 0.012, n = 21) (Figure 4B, D). In contrast, TC-2559 (2 μM) had no significant effect on sIPSC amplitude (34.92 ± 4.59 pA *vs *49.32 ± 11.60 pA, before and during TC-2559 perfusion, respectively) (*P *= 0.090, n = 21) (Figure 4C, E).

**Figure 5:**
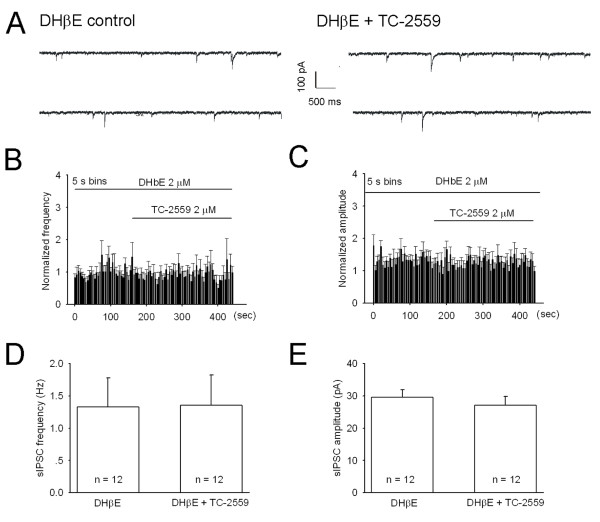
**TC-2559 enhances sIPSCs by activation of α4β2 subtype of nAchRs**. ***A***, Sample traces of sIPSCs recorded from a lamina II neuron in the presence of 15 μM DNQX and 2 μM DHβE before (left) and during bath application of 2 μM TC-2559 (right). Membrane potential was clamped at -70 mV. ***B-C***, Normalized rate histogram showing the sIPSC frequency (B) and amplitude (C) before and during bath application of 2 μM TC-2559 in the presence of 2 μM DHβE (n = 12). ***D-E***, In the presence of 2 μM DHβE, 2 μM TC-2559 had no significant effects on both the frequency (***D***, *P *= 0.6865, n = 12) and amplitude (***E***, *P *= 0.0978, n = 12) of sIPSCs (Paired *t *test).

DHβE (2 μM) applied alone had no effect on both sIPSC frequency and amplitude (99.7 ± 6.0% and 104.5 ± 9.9% of the control value, respectively; n = 4), but completely blocked TC-2559 (2 μM) induced sIPSC frequency enhancement (1.33 ± 0.36 Hz *vs *1.36 ± 0.38 Hz, before and during TC-2559 perfusion, respectively) (*P *= 0.687, n = 12) (Figure 5).

In addition, we also tested the effects of 10 μM TC-2559 on sIPSCs and the result was similar as that of 2 μM TC-2559. Bath application of TC-2559 (10 μM) increased the frequency of sIPSCs in 8 out of 10 (80%) neurons tested. As a whole group, sIPSC frequency were increased from 1.39 ± 0.39 Hz of the baseline control to 2.10 ± 0.63 Hz during TC-2559 perfusion (*P *= 0.041, n = 10), but without significant effect on the sIPSC amplitude (28.21 ± 7.48 pA *vs *34.67 ± 7.21 pA, before and during TC-2559 perfusion, respectively) (*P *= 0.200, n = 10).

These results indicate that acute application of TC-2559 enhanced inhibitory neurotransmission on spinal dorsal horn neurons by activation of α4β2 nAChRs.

### TC-2559 had no significant effect on excitatory synaptic transmission in superficial dorsal horn neurons

To test whether activation of α4β2 nAChRs by TC-2559 had any effect on excitatory neurotransmission within spinal dorsal horn, we then performed whole-cell patch-clamp recordings of spontaneous excitatory postsynaptic currents (sEPSCs) in superficial dorsal horn neurons in rat spinal cord slice. Membrane potential was voltage clamped at -70 mV.

**Figure 6:**
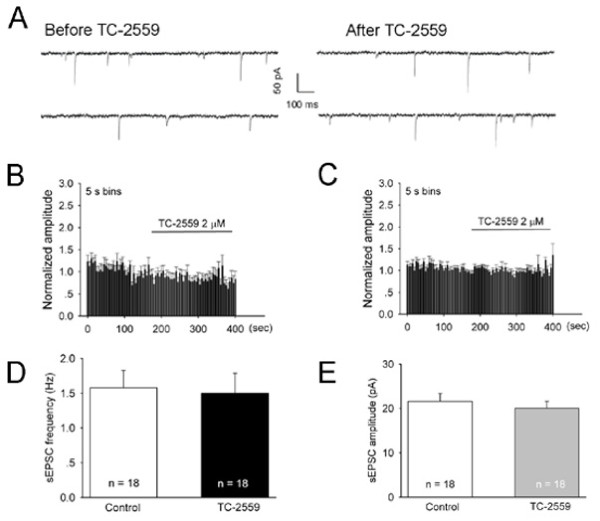
**TC-2559 has no effect on sEPSCs in the superficial dorsal horn of the spinal cord slices**. ***A***, Whole-cell voltage-clamp (V_h _= -70 mV) recording of sEPSCs in a SG neuron before and after bath application of 2 μM TC-2559. ***B-C***, Normalized rate histogram showing the mEPSC frequency (B) and amplitude (C) before and during bath application of 2 μM TC-2559 (n = 18). ***D-E***, 2 μM TC-2559 had no significant effect on both the frequency (***D***, *P *= 0.3065, n = 18) and amplitude (***E***, *P *= 0.7442, n = 18) of sEPSC (Paired *t *test).

Bath application of TC-2559 (2 μM) had neither effect on the sEPSC frequency (99.9 ± 8.2% of the baseline control) (*P *= 0.912, n = 18) nor on the sEPSC amplitude (95.1 ± 4.3% of the baseline control) (*P *= 0.196, n = 18), as shown in Figure 6. These results indicate that acute local activation of α4β2 nAChRs by TC-2559 has no effect on excitatory neurotransmission in neurons in the spinal superficial dorsal horn.

## Discussion

The animal pain behavior results from the present study suggest that selective α4β2 nicotinic receptor agonist TC-2559 has analgesic property, and *in vitro *spinal cord slice patch clamp recording results indicate that this analgesic property is probably medicated by selective enhancement of inhibitory neurotransmission in the spinal dorsal horn via activation of α4β2 subtype of nicotinic receptors. This conclusion is based on the observation that: 1) In *in vivo *animal pain models, TC-2559 inhibited both intraplantar formalin injection induced biphasic nociceptive responses and CCI-induced mechanical allodynia; 2) In *in vitro *spinal cord slices, TC-2559 significantly enhanced inhibitory synaptic transmission, but has no effect on the excitatory synaptic transmission.

### Bioavalaibility of TC-2559

Similar to what previously reported [16], brain exposure data of TC-2559 was obtained first as a guidance for our later *in vivo *and *in vitro *studies. The present brain exposure result demonstrated that TC-2559 at 3 mg/kg (i.p.) produced an initial peak whole brain concentration of about ~4 μM (15 min after injection), which quickly declined to ~2 μM at the 30 min point. The elimination of TC-2559 was fast with a half life of ~20 min which was similar to that of given by i.v. shown previously [16]. Since cerebral-spinal fluid is similar, we assumed that the spinal concentration of the TC-2559 after i.p. administration would be as the same as in brain. TC-2559 has been recently developed and characterised as a neuronal nAChR agonist [13] and selective for α4β2 subtype both *in vitro *[14,16] and *in vivo *[14,16]. The EC_50 _for TC-2559 to evoke excitation in VTA slice was found to be of 0.6 μM and TC-2559 is ineffective on α7 and α3β2 receptors up to 10 μM, and weakly effective on α2β4, α4β4 and α3β4 receptors, with EC_50_'s in the range of 10 - 30 μM [14]. Thus, at the 3 mg/kg i.p. dose for rat CCI study and 2 μM dose for *in vitro *brain slice experiment used in this study, TC-2559 is likely to be selective for the α4β2 nicotinic receptor subtype.

### Antinociceptive effects of TC-2559 in neuropathic pain animal models

Pain initiated or caused by a primary lesion or dysfunction in the nervous system is called neuropathic pain. Several animal models of neuropathic pain based on the induction of neuropathic pain behaviors after induction of a controlled nerve injury have been developed [20-22]. The formalin test is a model of injury-produced pain and is well established for its easier to perform and to standardize [23,24]. The intraplantar injection of formalin results in lifting, licking or other agitation behavior during an early phase, which resembles acute pain, followed by a late phase representative for neuropathic pain [24] which is comparable to CCI model. In current study, we demonstrated that TC-2559 dose-dependently reduced intraplantar formalin injection induced both early and late phases of nociceptive behaviors in mice. The dose-dependent inhibition on the late phase especially suggested that TC-2559 exerts antinociception effects on neuropathic pain. Nicotine itself has been demonstrated to have dose dependent antinociceptive effects in both the early and late phases of the formalin test in mice [25-27], and these effects were suggested to act via α4β2 subtype of nAChRs by using selective α4β2 nAChR antagonists. Thus, our results directly demonstrated for the first time that activation of α4β2 subtype of nAChRs by selective agonist could reduce the pain behavior induced by formalin in mice.

CCI model, which was first set up by Bennett in 1988, possesses the character of both peripheral and central sensitization [20]. It shares many common way with the clinical disease of neuropathic pain, and thus has been widely used in the field of pain study [28]. To further examine the antinociceptive action of TC-2559 on neuropathic pain, we then tested the effect of TC-2559 on CCI model in rat. In current study, in order to standardize the CCI model for our pharmacological study, the rats used for this study were selected initially by their pain threshold level during the pre-surgery pain behavioral test. Only those with a basal pain threshold between 6 and 10 g were chosen for CCI model surgery. As predicted, 4-7 days after CCI induction, 35% of the rats showed typical sensitized pain threshold under mechanical stimulation to be lower than 2 g in ipsilateral foot similar as previously reported [19]. Under this condition, we demonstrated that TC-2559, at the dose known selective for α4β2 subtype of nAChRs *in vivo *[12,13], reversed CCI-induced mechanical allodynia in all the rats tested. This result is the first time to show the analgesic role of α4β2 subtype of nAChRs in the rat CCI model. In addition, our data also showed that the analgesic effect of TC-2559 was quickly returning to the control level after injection, which is consistent to our ADME result of the property of TC-2559 *in vivo*. Our bioavailability study showed that TC-2559 was a compound with fast absorption and quick elimination properties. The *in vivo *half life in both brain and serum for TC-2559 was only about 20 min, which was well reflected for its short analgesic action *in vivo *in our current study. Thus, our *in vivo *results demonstrated in two commonly used animal pain models and in addition, in two different species (mouse and rat) that TC-2559 is a potent antinociceptive compound.

In contrast to the antinociceptive action of TC-2559 on injured hindpaw at the doses of 1 and 3 mg/kg, TC2559 at the dose of 1 mg/kg had no effect on the paw withdrawal threshold in the contralateral hind paw. However, when the drug dose was increased to 3 mg/kg, TC-2559 significantly increased the paw withdrawal threshold in the contralateral hind paw, along with on ipsilateral hindpaw, compared to that of vehicle control. This effect suggests that over activation of α4β2 subtype of nAChR by using high dose of TC-2559 may suppress the basal sensation of spinal cord sensory transmission. It is consistent with the results obtained in a previous study, where a low dose of nicotine (10 nmol i.t.) or epibatidine (0.3 nmol i.t.) has no effect in sham animals but is antiallodynic in peripheral nerve injury mice [11]. Both results suggest that the injured peripheral nerve is more sensitive to nicotinic receptor activation, which may indicate of an upregulation of nicotinic receptors after injury. This has given a therapeutical window to reduce sensitized pain threshold for nicotinic drugs such as TC-2559. Since the analgesic effect of TC-2559 on ipsilateral hindpaw was observed at the dose as low as 1 mg/kg, our current results indicated that the therapeutic window for TC-2559 on pain release might be likely between 1 and 3 mg/kg (i.p.) doses in rats. It suggests that the ligand acts as selective α4β2 subtype of nAChR agonist may be developed to have a clinically acceptable therapeutical window between pain signal sensitivity and normal sensory signal transmission.

DHβE, a widely used compound served as a selective α4β2 nAChR antagonist [12,13], reversed the antinociceptive action of TC-2559 in rat CCI model in this study, while DHβE applied alone had no significant effect on CCI-induced mechanical allodynia. These results suggest that the antinociceptive effect of TC-2559 on neuropathic pain is indeed mediated by activation of α4β2 subtype of nAChRs. In several previous studies in rats, intrathecal injection of DHβE alone had no effects while they blocked the nociceptive or antinociceptive effects of the nicotinic agonists [29,30]. These are consistent with our present observation. In addition, in our *in vitro *studies in the spinal cord slices, DHβE alone also had no significant effect on sIPSCs, which is consistent with our *in vivo *experiments.

However, the lack of effect of DHβE in rats seems contradictory with studies performed on mice. Rashied et al. demonstrated that bath application of DHβE induces a decrease in the frequency of IPSCs in two third of dorsal horn neurons in mice [12]. Also, i.t. injection of DHβE induces mechanical allodynia in mice [11,12]. These differences in mice and rats are likely due to species differences. We hypothesis that the Ach release level in mice might be higher than that in rats. However, we choose to perform behavior experiments on different species of animals is because this study was designed to test whether the selective agonist of α4β2 subtype of nAChR (TC-2559) has antinociceptive effects and its possible mechanism, thus to test whether the agonist of α4β2 subtype of nAChR is capable to be developed as new common analgesic drug for treatment of neuropathic pain. It would be better to know whether TC-2559 could have analgesic effects on species known to have differences since finally it may be used on human beings. Therefore, we choose to perform behavior experiments (formalin and CCI) on different species of animals. Our results of formalin test and CCI demonstrated that TC-2559 has analgesic effects on both mice and rats, despite they have differences.

Thus, our current study is the first to demonstrate, by using selective α4β2 nAChR agonist (TC-2559), that direct stimulation of α4β2 subtype of nAChRs has strong antinociceptive effects in classic neuropathic pain animal models.

### Spinal mechanisms of antinociceptive effects of TC-2559

The inhibitory interneurons in the spinal dorsal horn are crucial for maintaining normal input intergration and are localized throughout the spinal dorsal horn [31,32]. The neuronal pathways mediating allodynia/hyperalgesia in spinal dorsal horn are normally under strong inhibitory regulation [33-35]. Since α4β2 subtype of nAChRs were reported to be preferentially expressed in the inhibitory interneurons in the spinal dorsal horn [36], we hypothesized that activation of α4β2 subtype of nAChRs by TC-2559 would excite those inhibitory interneurons expressing α4β2 nAChRs. In turn, the pain input sensory pathway between primary afferent fibers and lamina I nociceptive specific neurons in spinal dorsal horn would receive more inhibitory control after activation of α4β2 subtype of nAChRs. Indeed, our results showed that TC-2559, a selective α4β2 nAChR partial agonist [14], significantly enhanced the sIPSC frequency in majority of the spinal superficial dorsal horn neurons tested. This enhancement could be fully antagonized by a selective α4β2 nAChR antagonist (DHβE) indicated that TC-2559 was indeed activated α4β2 nAChRs in spinal dorsal horn. These results suggest that activation of α4β2 nAChRs would generally increase the inhibitory tone in the spinal dorsal horn, thus may regulate both the sensory input and signal integration in this region.

Hyperalgesia is mediated, in spinal cord level, by enhanced pain signal transmission and reduced local inhibition [35,37,38]. However our data showed that TC-2559 by activation of α4β2 nAChR had no significant effect, in contrast to increase of inhibitory transmission, on the excitatory neurotransmission in spinal dorsal horn neurons. This result is consistent with a previous report that nicotine enhances excitatory synaptic transmission in the spinal dorsal horn by α7 [39] but not by α4β2 nAChRs. This is further supported by the finding that α4β2 subtype of nAChRs expressed preferentially in the inhibitory interneurons in the spinal dorsal horn [36]. Thus, as expected, activation of these receptors would have predominant effect on the inhibitory, rather than excitatory neurotransmitter release in this region. This result suggests that the antinociciptive effect of TC-2559, by activation of α4β2 nAChRs, is likely due to solely modulation of the inhibitory circuits within the spinal dorsal horn to enhance the basal inhibition, but not affecting either excitatory sensory input or excitatory interneuron function.

Although the methods that by patch clamp recording on visually identified spinal superficial dorsal horn neurons could not resolve cell identity, the high prevalence of enhancement on sIPSCs by TC-2559 (~85% of neurons with > 20%of increase on sIPSC frequency) suggests that this type of inhibitory modulation in spinal superficial dorsal horn might be common to multiple populations of the neurons in this region of the spinal cord. This is consistent with the action of nicotine with which GABAergic synaptic transmission was enhanced in 86% of neurons tested in the spinal dorsal horn [40]. Thus it suggests that TC-2559 would enhance the general inhibition in the superficial dorsal horn neurons by activation of α4β2 subtype of nAChRs.

Although our results clearly demonstrated that TC-2559 by activation of α4β2 nAChRs to enhance the basal inhibition in spinal dorsal horn neurons which in turn, as expected, to reduce the pain related peripheral sensory input at the spinal cord level, we can not rule out the possibility of the involvement of the activation of α4β2 nAChRs localized in the regions other than spinal cord dorsal horn, while tested in *in vivo *pain animal models by systemic application of TC-2559. However, since spinal dorsal horn is an important pain signal primary integration region, we hypothesis that activation of α4β2 nAChRs localized in the spinal dorsal horn by TC-2559 to enhance the over all inhibitory tone is one of the major contributor for TC-2559's antinociciptive action.

### Functional implication

Spinal dorsal horn neurons including those mediating allodynia/hyperalgesia signals are normally under strong inhibitory regulation [33-35]. And any mechanism to release those inhibition on the pain signaling pathway in the spinal dorsal horn opens the gate to pathological pain sensory input to spinal dorsal horn neurons [33,35,41], which would cause the chronic pain hypersensitivity [11]. Reports have shown that disruption of this inhibition by blockade of α4β2 subtype of nAChRs induced thermal and mechanical hyperalgesia in both normal rats [29,30] and naive mice [12], which indicates that α4β2 subtype of nAChRs play a crucial modulatory role to maintain the tonic inhibition in spinal dorsal horn for sensory and/or pain signal input. The data from our current study were indeed showing that direct activation of α4β2 subtype of nAChRs by agonist of TC-2559 did exert antinociceptive effects in pathological pain animal models of both formalin test and CCI possibly by enhancing the inhibitory neurotransmission in spinal dorsal horn. In addition, we showed that TC-2559, acting as a selective α4β2 subtype of nAChR agonist, deferentially enhanced inhibitory neurotransmission in spinal dorsal horn neurons but not affecting excitatory neurotransmission. This property gives the hope that α4β2 nAChRs agonist based analgesic drug might have fewer side effects than that of currently marketed pain relieves, in terms of the spinal cord level. However, when given intracerebroventricularly (i.c.v.) and intracisternally (i.c.), TC-2559 has been reported to induce an increase in blood pressure and renal nerve activity [42]. What's more, we have also previously demonstrated that TC-2559, while given intravenously, activates dopaminergic neurons in the VTA and suppress hippocampal long term potentiation [29,30] in anaesthetized rats. Thus TC-2559 itself is therefore likely to have numerous physiological functions beside analgesia. Since TC-2559 is an selective partial α4β2 nAChR agonist [29,30], developing more potent agonist selective for α4β2 nAChRs may be one of the directions for developing nicotinic receptor based analgesic drug.

## Conclusions

The antinociciptive property of α4β2 subtype of nAChRs has been previously reported with the aid of the selective antagonist [12,29,30]. Here, we demonstrated for the first time by using a selective α4β2 subtype of nAChR partial agonist TC-2559, that direct stimulation of the α4β2 nAChRs has analgesic effect on pathological pain. Our behavioral and electrophysiological data indicated that TC-2559 exerts antinociception effects on pathological pain models, and enhancement of spinal dorsal horn inhibitory neuronal transmission via activation of α4β2 subtype of nicotinic receptors is one of the underlying mechanisms. Although the ADME property (fast elimination rate), the narrow therapeutic window and numerous other physiological function beside analgesia makes TC-2559 with limited drug ability, our results are still the first direct evidence to strongly support the notion that the agonist of α4β2 subtype of nAChR is capable to be developed as new analgesic drug for treatment of neuropathic pain with less side effects.

## Methods

### Experimental animals

Experiments were performed on adult male mice (body weight 15-30 g), adult male (body weight 200-220 g) and young (postnatal days 14-21) SD rats for formalin test, CCI, brain exposure and *in vitro *electrophysiological experiments, respectively. All the animals were purchased from Shanghai Experimental Animal Center, Chinese Academy of Sciences, Shanghai. Animals were kept on a 12 hour light: dark *cycle *with a room temperature of 23 ± 1°C and received food and water *ad libitum*. All procedures of the experiments were approved by the Committee of Animal Use for Research and Education of Fudan University, and all efforts were made to minimize the number of animals used and their suffering, in accordance with the ethical guidelines for animal research.

### Brain exposure experiments

A total of 8 male Sprague Dawley rats were used in these experiments. Rats were killed by overdose of urethane (5 g/kg, i.p.) at different time points (0.25, 0.5, 1 and 2 h) after TC-2559 (3 mg/kg, i.p.) administration. Brain tissues were quickly removed for *in vitro *measurements, similar as previously reported [12]. Brain samples were prepared by the addition of twice the volume (ml) of water to weight (g) of brain cortex, followed by homogenisation to produce a smooth suspension. Homogenised brain cortex samples (500 μl) were then extracted with methyl-t-butyl ether and centrifuged. The solvent layer was transferred to clean tubes and evaporated under nitrogen at 401C. The residue was dissolved in 50% methanol and analysed using liquid chromatography and a Sciex API 3t Mass Spectrometer, with turbo-ionspray in positive ion MRM mode, for detection. Data were calculated using MacQuan 1.4. In order to estimate the total brain concentration, 1 g of the brain tissue was assumed to be equivalent to be 1 ml in volume.

### Formalin test

Classic formalin test was performed as previously described [18,43]. Briefly, after 15 minutes of stabilizing period in an experimental beaker, mice received intraplantar injection of 5.0% formalin solution (50 μl, saline vehicle) to the right hindpaw, and placed back to the beaker immediately for nociceptive behavior observation. Either vehicle (saline) or different doses of TC-2559 (1, 3, 10 mg/kg) were administered intraperinatally 5 min before formalin injection. Animals were sacrificed immediately at the end of the study by the intraperinatal injection of over dose of urethane and neck displacement.

Nociceptive behavior elicited by formalin injection, such as lifting, licking and biting of the injured paw were recorded and timed for every 5-minute time bins manually for 45 minutes continuously. The formalin pain score was calculated using the following formula as previous reported [43]: formalin pain score = [seconds spent on paw lifting + 2 × (seconds spent licking or biting injected paw)]/300. Data were then analyzed for early (0-10 mins) and late (11-45 mins) phases as previous reported [43].

### Chronic constriction injury (CCI) of the sciatic nerve and the measurement of mechanical allodynia

Rats were deeply anesthetized with 2% pentobarbital sodium (40 mg/kg, i.p.), judged by absence of paw withdraw reflex. CCI operation was performed as previously described [19] in a sterilized environment. After the surgery, animals were retuned to their home cages for recovery, for at least 5 days. The paw withdrawal threshold (PWT) was determined using a calibrated series of von Frey hairs (Stoelting, IL, USA) ranging from 1 to 26 g. Before the behavioral tests, animals were allowed to acclimate for 30 min in the animal facilities. A series of 9 calibrated von Frey hairs were applied in ascending order (1, 1.4, 2, 4, 6, 8, 10, 15 and 26 g). A particular hair was applied until it buckled. This was maintained for approximately 2 sec. A trial consisted of application of a von Frey hair to the hind paw five times at 5-sec intervals. If withdrawal did not occur during five application of a particular hair, the next larger hair in the series was applied in a similar manner. When the hind paw was withdrawn from a particular hair in three out of five consecutive applications, the value of that hair in grams was considered to be the withdraw threshold. After the threshold was determined for one hindpaw, the same testing procedure was repeated on the other hindpaw at 5-min interval. The rats received continued measurement of PWT for 3 days before CCI surgery. Rats with PTW at between 6 g - 10 g for both paws were chosen for CCI operation in this study. Assessment of PWT began 5 days after surgery. Either drugs or vehicle were tested only when the PTW after CCI reached to a stable level as shown in Figure 2A. In drug testing day, PWT was measured once 10 min before TC-2559 at different doses or saline injection (i.p.), and 3 times after drug administration at 15 min inter test interval.

### Preparation of spinal cord slices

As we previously described [44], young SD rats (postnatal days 14-21) were deeply anesthetized with diethyl ether, and for spinal nerve blocking, about 1 ml lidocaine (5 ml: 0.1 g) was injected to both sides of lumbar vertebrae (L4-5). Laminectomy was performed from mid-thoracic to low lumbar levels and the cord was quickly removed to cold modified artificial cerebrospinal fluid (ACSF), which was containing (in mM): NaCl, 80; KCl, 2.5; NaH_2_PO4, 1.25; CaCl_2_, 0.5; MgCl_2_, 3.5; NaHCO_3_, 25; sucrose, 75; ascorbate, 1.3; sodium pyruvate, 3.0 with pH at 7.4 and osmolarity at 310.5 mOsm, and oxygenated with 95% O_2 _and 5% CO_2_.. Transverse 500 μm slices were obtained. Slices were then incubated for at least 1 h at 35°C in a solution that consisted of: (in mM) NaCl, 125; KCl, 2.5; CaCl_2_, 2; MgCl_2_, 1, NaH_2_PO4, 1.25; NaHCO_3_, 26; D-glucose, 25; ascorbate, 1.3; sodium pyruvate, 3.0 with pH at 7.4 and osmolarity at 324.5 mOsm, and oxygenated with 95% O_2 _and 5% CO_2_. The slice was then transferred into a recording chamber and perfused with oxygenated recording solution at a rate of 5 ml min^-1 ^prior to electrophysiological recordings at room temperature.

### Whole-cell patch clamp recordings from spinal cord slices

Neurons were identified by infrared differential interference contrast (IR-DIC) video microscopy with an upright microscope (Nikon, LV-TV) equipped with a 40×, 0.80 NA water-immersion objective and a CCD camera (IR-1000E, USA). Patch pipettes (5-10 MΩ) from borosilicate glass (Sutter Instruments, Novato, CA, USA) were made on a horizontal micropipette puller (P-97, Sutter Instruments, Novato, CA, USA). The pipettes used for recording inhibitory currents were filled with (in mM): 140 CsCl, 2 MgCl_2_, 1 CaCl_2_, 1 EGTA, 10 HEPES, 5 K-ATP, 0.1 GTP, pH = 7.4. Using these solutions, at the holding potential of -70 mV, inhibitory currents appear as inward deflection. When recording sIPSCs in the superficial laminae of the spinal dorsal horn neurons, 15 μM DNQX was included in the external bath solution to block non-NMDA receptor glutamatergic currents. The pipette solution used for recording excitatory currents contained (in mM): potassium gluconate 120, KCl 20, MgCl_2 _2, Na_2_ATP 2, NaGTP 0.5, HEPES 20, EGTA 0.5, pH 7.28 with KOH, measured osmolarity 300 mM. Data were acquired using an MultiClamp 700B patch-clamp amplifier (Axon Instruments, USA). Access (series) resistances of all cells were monitored and recorded every one minute throughout the experiment by applying a 2 mV hyperpolarizing pulse for 2 ms. Only those cells that showed < 20% change in access resistance over the period of experiments were included in the data analysis. Responses were low-pass filtered on-line at 2 kHz, digitized at 5 kHz, and analyzed off-line using Clampfit 10.0 and Mini 60 software. Membrane potential was held at -70 mV and all recordings were performed at room temperature. Drugs were bath applied by exchanging perfusion solution containing a known drug concentration without altering the perfusion rate and the time period for drug reach the recorded spinal slice was estimated at ~40s. IPSCs or EPSCs recorded 1 min before drug application were served as baseline control, and drug effects were assessed during the second minute after drug application. When normalized, data were expressed as % of control. The drug effect on either IPSCs or EPSCs was defined as "increase"or "decrease" if the variation compared with control were ± 20%, respectively, as previous reported [42].

### Reagents

Dihydro-β-erythroidine (DHβE), bicuculline methiodide, dihydro-h-erythroidine hydrobromide 6,7-dinitroquinoxaline-2,3-dione (DNQX), strychnine, Ethylene glycol-bis(2-aminoethylether)-*N, N, N', N'*-tetraacetic acid (EGTA), HEPES, K-ATP and Mg-GTP were all purchased from sigma. TC-2559 was a gift from Eli Lilly Company. For *in vivo *studies, TC-2559 and DHβE were dissolved in normal saline. For *in vitro *experiments, all of the drugs except DNQX (initially dissolved in dimethylketone) were dissolved in deionized water and prepared as stock solutions and diluted to the required concentration (1:1000) with ACSF. All drugs were bath applied in *in vitro *experiments.

### Data analysis and statistics

Data were expressed as mean ± s.e.m. Effects of drugs on behavior tests between two groups were compared with two-way ANOVA followed by the Holm-Sidak test for time course measures and *t*-test for point-to-point comparison. Paired *t*-test or unpaired *t*-test was used for data from *in vitro *electrophysiology experiments. *P *< 0.05 was considered statistically significant.

## List of abbreviations

(ACSF): Artificial cerebrospinal fluid; (CCI): Chronic constriction injury; (DHβE): Dihydro-h-erythroidine; (DNQX): Dihydro-h-erythroidine hydrobromide 6,7-dinitroquinoxaline-2,3-dione; (EGTA): Ethylene glycol-bis(2-aminoethylether)-*N, N, N', N'*-tetraacetic acid; (nAChR): Nicotinic acetylcholine receptor; (NSAID): Nonsteroidal anti-inflammatory drug; (sEPSC): Spontaneous excitatory postsynaptic current; (sIPSC): Spontaneous inhibitory postsynaptic currents.

## Competing interests

The authors declare that they have no competing interests.

## Authors' contributions

LZC and LH performed electrophysiological experiments in the spinal cord slice and drafted the manuscript. JF and LTH performed the *in vivo *experiments. YW and LCP designed the experiments and revised the manuscript. All authors have read and approved the final manuscript.
